# A 4-year-old child presenting morning onset of spontaneous tracheal rupture due to bronchial mucous plug occlusion during the nighttime sleep: a case report

**DOI:** 10.1186/s13256-016-0912-9

**Published:** 2016-06-01

**Authors:** Raffaella Capasso, Mattia Carbone, Eugenio Rossi, Rosanna Mamone, Raffaele Zeccolini, Alfonso Reginelli, Massimo Zeccolini, Luca Brunese, Antonio Rotondo

**Affiliations:** 1 Department of Internal and Experimental Medicine, Magrassi-Lanzara, Second University of Naples, Piazza Miraglia 2, 80138 Naples, Italy; 2 Department of Radiology, A.O.U. San Giovanni di Dio e Ruggi d’Aragona, Via San Leonardo, 84131 Salerno, Italy; 3 Department of Radiology, Santobono-Pausilipon-Annunziata Children’s Hospital, Via Posillipo 226, 80123 Naples, Italy; 4 Faculty of Medicine and Surgery, Second University of Naples, Via Costantinopoli 16, 80138 Naples, Italy; 5 Department of Health Science, University of Molise, Via De Sanctis, 86100 Campobasso, Italy

**Keywords:** Tracheal rupture, Mucous plug, Cough reflex, Atelectasis

## Abstract

**Background:**

Coughing is the most efficient mechanism for clearing mucus and fluid secretions from the airways and its reflex can be suppressed by sleep. Spontaneous tracheal ruptures are believed to result from raised intratracheal pressure against a closed glottis, such as for severe coughing. This is the first reported case of tracheal rupture presented on morning awakening after bronchial mucous plug formation during the nighttime sleep because of an ineffective cough reflex.

**Case presentation:**

An otherwise healthy white 4-year-old child presented morning onset of dyspnea, chest pain and diffuse swelling of the neck. His history was significant only for nonsevere coughing episodes before his nighttime rest; the child’s parents denied any recent fever, weight loss, pains, trauma, bronchial asthma, and sick contacts. A chest X-ray and computed tomography scan revealed pneumomediastinum, obstructive atelectasis of the lower lobe of his left lung, and a small tracheal laceration confirmed by an emergency bronchoscopy. After endoscopic removal of a mucous plug and secretions, the child’s pulmonary gas exchange and respiratory rate improved, so our patient was managed conservatively.

**Conclusions:**

This report illustrates an unusual presentation of lung obstructive atelectasis due to a mucous plug manifested by tracheal rupture. This report also highlights the importance of the coughing reflex as one of several defensive mechanisms protecting the airways from the potentially damaging effects of aspirate and accumulated secretions.

## Background

Tracheal rupture (TR) is a rare and potentially life-threatening injury in both adults and children [1, 2]. In adults it is usually caused by endotracheal intubation, cervical or thoracic trauma, but spontaneous tracheal lacerations have been reported in association with chronic steroid usage, acute bronchitis, external beam radiation therapy, and acquired tracheomalacia [1, 2]. In children spontaneous TR is extremely uncommon with only a handful of published cases [1]. To the best of our knowledge, this is the first report of lung obstructive atelectasis due to mucous plug formation during the nighttime sleep and manifesting as morning-onset spontaneous TR.

## Case presentation

**Fig. 1:**
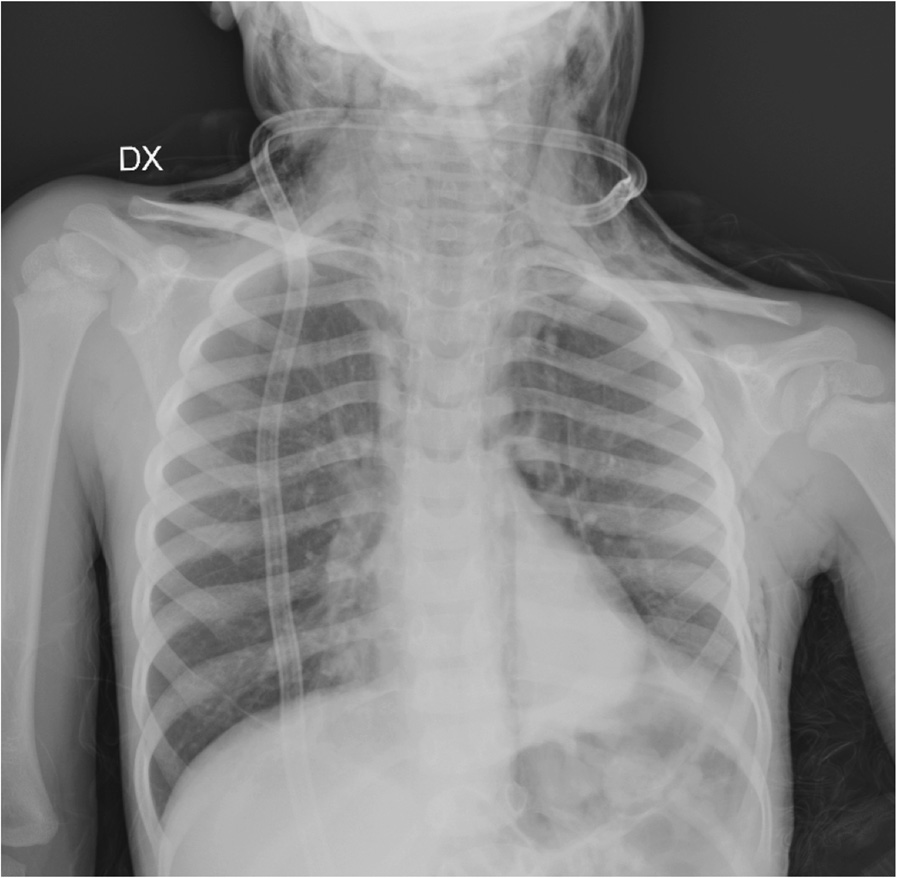
Chest X-ray examination revealed the presence of diffuse subcutaneous emphysema of the neck extending along the left lateral chest wall. Pneumomediastinum was also appreciable: air within the superior mediastinum on both sides and along the left paravertebral space. A loss of volume of the left lung due to left inferior lobe atelectasis was associated with the elevation of the left diaphragmatic dome

**Fig. 2:**
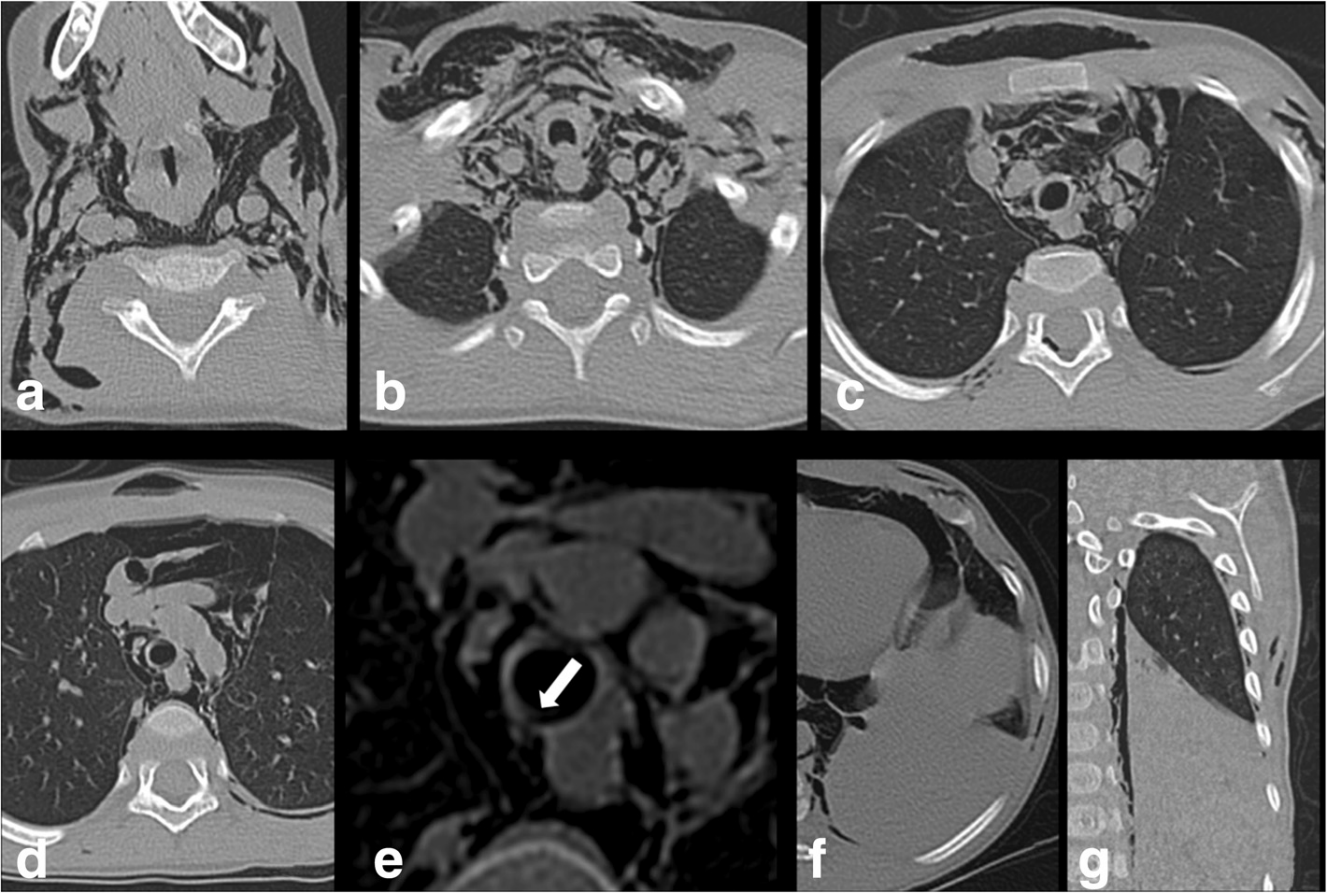
Computed tomography scan of the thorax showed subcutaneous soft tissue emphysema and air dissecting through the neck and mediastinal spaces (**a,b**). Minimal pneumothorax (**b–d**), pneumorrhachis within the extradural space in the upper thoracic spine (**c**), and pneumopericardium (**d**) were also noted. Thin laceration of the right posterolateral wall of the trachea was detectable (**e**, *arrow*). Axial and coronal views showed reduction of volume and absence of air bronchogram of the lower lobe of the left lung (**f,g**)

**Fig. 3:**
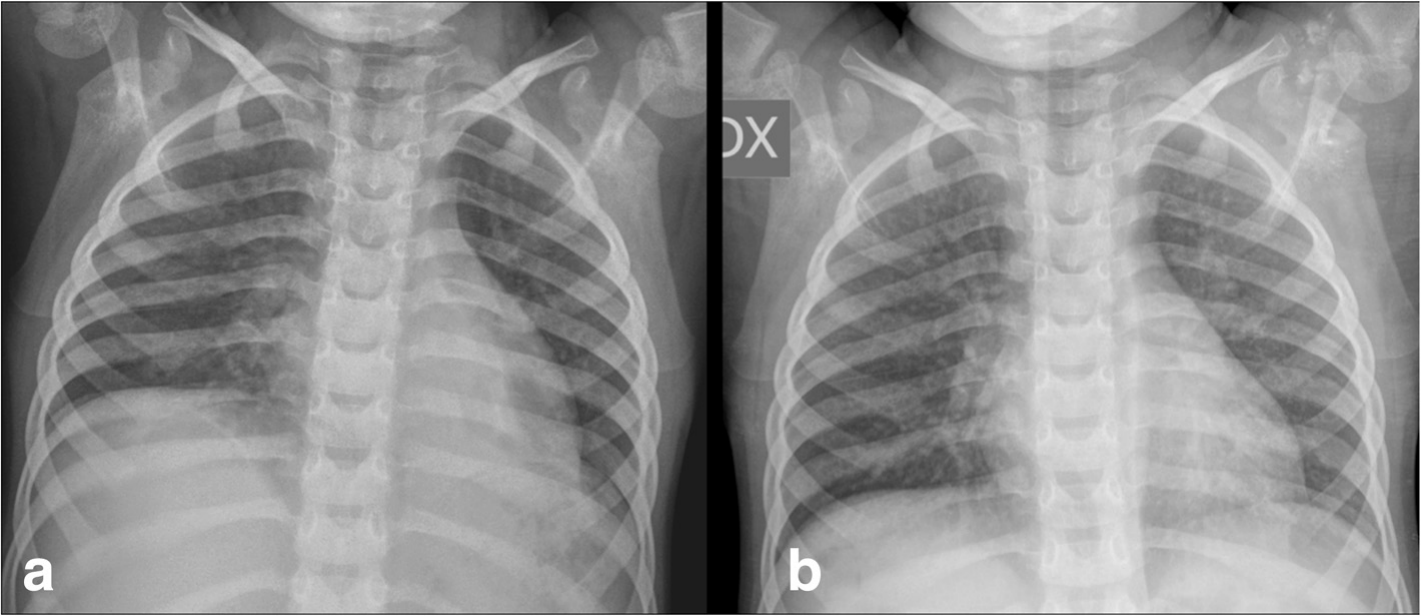
Chest X-rays respectively performed 12 days (**a**) and 5 weeks (**b**) after admission showed the rapid complete absorption of air collections, the improved left lung expansion, and the gradual resolution of the retrocardiac atelectasis with air bronchogram reappearance (**b**)

A 4-year-old white boy was brought by his parents to the emergency department of our hospital with complaints of diffuse cervical swelling and chest pain. His history was significant only for nonsevere coughing episodes before his nighttime rest; the child’s parents denied any recent fever, weight loss, pains, trauma, bronchial asthma, and sick contacts. An examination revealed tachypnea, shallow breathing, decreased breath sounds especially on the left posterior field, and palpable subcutaneous emphysema with crepitus on both sides of his neck. The child was dyspnoeic but not cyanotic, while his oxygen saturation progressively decreased. Chest radiography (CXR) displayed cervical subcutaneous emphysema, signs of pneumomediastinum, a hyperinflated right lung, and triangular atelectasis of the lower lobe of his left lung behind his heart (Fig. 1). A computed tomography (CT) scan of his thorax confirmed and better depicted the CXR findings (Fig. 2a, b), revealed the presence of pneumopericardium, pneumorrhachis and pneumothorax, air in the retropharyngeal and paraesophageal spaces (Fig. 2b–d), and showed a 8 mm longitudinal laceration in the right posterolateral wall of his lower trachea (Fig. 2e), 19 mm above the carina, associated with a nearly total obstructive atelectasis of the lower lobe of his left lung without detectable foreign bodies (Fig. 2f, g). The air collections were considered to have originated from the tear in the tracheal membrane. Because his symptoms worsened, the child was intubated, given intravenous fluids and vasopressors to control the hypotension, and was transferred to the pediatric intensive care unit of another institution. He underwent an emergency bronchoscopy, which confirmed the tracheal injury and disclosed abundant thick secretions with a large mucous plug blocking the lower lobe bronchial lumens of his left lung. Soon after the aspiration of fluids and the removal of the mucous plug, the child’s pulmonary gas exchange and respiratory rate improved, and his left lung expansion increased. There were neither underlying endobronchial lesions nor infections. The child was managed with conservative treatment consisting of 5 days of intubation and administration of intravenous corticosteroids, broad-spectrum antibiotics, and inhaled bronchodilators. Daily physical and CXR examinations revealed rapid absorption of the subcutaneous emphysema and air collections, and gradual resolution of the atelectasis (Fig. 3). The child was discharged from the pediatric hospital 35 days after admission, and his follow-up was planned at that institution.

## Discussion

Spontaneous TR is a rare phenomenon in the pediatric population, but despite its rarity, it represents an important condition to be aware of in children because prompt appropriate treatment can be lifesaving [1, 2]. Spontaneous TRs are believed to result from raised intratracheal pressure against a closed glottis, such as for severe coughing, and occur especially at the lower third of the trachea and the cricothyroid membrane [1, 2]. The laceration usually extends longitudinally in the posterior membranous wall of the trachea or involves the junction between the membranous wall and cartilaginous ring [2]. In accordance with these considerations, the rupture in our case was located in the right lower third of the posterior membranous tracheal portion, although the child’s parents did not report severe coughing episodes. Coughing is the most efficient mechanism for clearing mucus and fluid secretions from the airways. The total amount of mucus in the conducting airways is determined by the rate of secretion and the clearance of mucus by epithelial reabsorption, evaporation, ciliary movement, and cough transport [3]. When coughing is weak, bronchial secretions can accumulate to the point that they obstruct airflow and mucous plugging occurs and, if it cannot be cleared, the situation can be serious, as in our case. [4]. The magnitude of the cough reflex is influenced by both the site of stimulation and the central nervous state [5]. Laryngeal and tracheal stimulation causes vigorous respiratory responses, while the irritation of bronchi, bronchioles, and alveoli determines little or no effective cough reflex [4, 5]. Studies of anesthetized humans have shown that the cough reflex is suppressed [4–6]. In accordance with this, some adult cases have been described in the English literature of sudden lung collapse or segmental acteletasis, with or without pneumomediastinum due to mucous plug formation, that occurred during and after general anesthesia because of weak cough reflex [7–9]. Similarly, sleep is also known to suppress the cough reflex, especially during rapid eye movement (REM) sleep, and some studies demonstrated that irritant stimuli can cause coughing only if the stimulus first produced arousal, but the biological mechanisms for this action are poorly understood [4, 5]. To the best of our knowledge, lung atelectasis due to mucous plug formation during nighttime sleep and manifesting as TR has not yet been described. In our case, the child’s parents did not notice nor complain of cough during the child’s nighttime sleep and, at the time of hospital admission, they recounted that the child slept well. However, before his nighttime rest our little patient had some weak coughing episodes, probably indicating an underlying irritative cause – that remained unknown – which could have produced mucous bronchial hypersecretion. We could hypothesize that during the nighttime, the collection of secretions in the peripheral airways and the sleeping state may have lead to ineffective cough reflex, so the child had an uninterrupted sleep probably without agitation and changes of decubitus. Thus the secretions became stagnant and accrued in the mainstream bronchi, forming a mucous plug. The mucous plug and the suppressed cough reflex played a synergetic effect, amplifying the extent of bronchial lumen obstruction, causing lower lobe atelectasis of his left lung. When the child awoke, the cough reflex resulted in a few effective and tussigenic paroxysmal attacks, not noticed by the child’s parents, which caused TR during the compressive coughing phase, when the glottis closes and forced expiration with a rapid rise in intrathoracic pressure takes place. Then the air leaking from a TR spread throughout in the soft tissue planes of his neck, retropharynx, pericardium, mediastinum, spinal canal, and pleural space, resulting in subcutaneous emphysema and respiratory distress at the moment of the child’s admission to our hospital. Starting from the first report of spontaneous TR in a 7-year-old boy with acute tracheobronchitis by Roh and Lee, only another four reports have been described in the English literature, including cases respectively associated with paroxysmal productive coughing in a 14-year-old boy, with presumptive anaphylaxis and no pathology revealed by bronchoscopy in a 3-year-old boy, with violent vomiting in a 4-year-old girl, and with a 2-day history of severe coughing in an 18-month-old boy [1, 2, 10–12]. To the best of our knowledge, this is the first reported case of spontaneous TR secondary to nocturnal obstructive atelectasis by secretions and mucous plug in an otherwise healthy child. Even though rare, TR should be considered the cause of respiratory distress when clinical examination and CXR reveal signs of nonextended lung obstructive atelectasis despite important air leakage in the soft tissue planes.

CXR is the initial imaging examination in the diagnostic process to also investigate the presence of pneumothorax, which is one of the initial presumptive diagnoses in case of neck and thorax subcutaneous emphysema, but it does not allow the direct detection of tracheal injuries. CT is more sensitive for detecting TR or related complications, for evaluating the extent and localization of air collections, and it also makes possible the assessment of underlying pulmonary disease. In our case, CT was able to demonstrate the presence of a short tracheal tear associated with complete lower lobe atelectasis of the left lung without air bronchogram. The definitive diagnosis should be reached by using fiberoptic endoscopy, which must be performed as soon as possible if there is any question of an airway rupture because TR is potentially lethal [2]. In our case, the longitudinal TR was diagnosed on both CT and endoscopic examination. Moreover, bronchoscopy showed mucoid impaction in the lower lobe of the left lung, allowed the removal of the mucous plug, and the aspiration of the bronchial secretions. Following this, the child had marked improvement in his oxygenation and ventilation, and his left lung expanded after the plug was removed. During follow-up, serial CXRs are considered to be sufficient for evaluating the resolution of the air collections, as in our case. Generally, localized short (<2 cm) lacerations can be treated conservatively with empiric broad-spectrum antibiotic therapy and intubation with the cuff inflated distally to the tear to prevent further air leakage through the injury site. Otherwise surgical repair is preferred [2]. In our patient, the tracheal longitudinal tear was short and the child’s clinical condition clearly improved after therapeutic bronchoscopy, consequently the TR was managed conservatively.

## Conclusions

This report describes an unusual but potentially lethal presentation of obstructive lung atelectasis due to mucous plug formation during the nighttime sleep. It also illustrates the importance of the coughing reflex as one of several defensive reflexes protecting the airways from the potentially damaging effects of aspirate and accumulated secretions.

## Abbreviations

CT: computed tomography
CXR: chest X-ray
TR: tracheal rupture
